# Myo-Inositol and D-Chiro-Inositol as Modulators of Ovary Steroidogenesis: A Narrative Review

**DOI:** 10.3390/nu15081875

**Published:** 2023-04-13

**Authors:** Mariano Bizzarri, Noemi Monti, Aurora Piombarolo, Antonio Angeloni, Roberto Verna

**Affiliations:** 1 Department of Experimental Medicine, Sapienza University, Via A. Scarpa 16, 00160 Rome, Italy; 2 Systems Biology Group Lab, Sapienza University, 00160 Rome, Italy

**Keywords:** myo-inositol, pyrophosphates, inositol phosphates, phosphoinositol phosphates, steroidogenesis, insulin resistance

## Abstract

Myo-inositol is a natural polyol, the most abundant among the nine possible structural isomers available in living organisms. Inositol confers some distinctive traits that allow for a striking distinction between prokaryotes and eukaryotes, the basic clusters into which organisms are partitioned. Inositol cooperates in numerous biological functions where the polyol participates or by furnishing the fundamental backbone of several related derived metabolites, mostly obtained through the sequential addition of phosphate groups (inositol phosphates, phosphoinositides, and pyrophosphates). Overall myo-inositol and its phosphate metabolites display an entangled network, which is involved in the core of the biochemical processes governing critical transitions inside cells. Noticeably, experimental data have shown that myo-inositol and its most relevant epimer D-chiro-inositol are both necessary to permit a faithful transduction of insulin and of other molecular factors. This improves the complete breakdown of glucose through the citric acid cycle, especially in glucose-greedy tissues, such as the ovary. In particular, while D-chiro-inositol promotes androgen synthesis in the theca layer and down-regulates aromatase and estrogen expression in granulosa cells, myo-inositol strengthens aromatase and FSH receptor expression. Inositol effects on glucose metabolism and steroid hormone synthesis represent an intriguing area of investigation, as recent results have demonstrated that inositol-related metabolites dramatically modulate the expression of several genes. Conversely, treatments including myo-inositol and its isomers have proven to be effective in the management and symptomatic relief of a number of diseases associated with the endocrine function of the ovary, namely polycystic ovarian syndrome.

## 1. Relevance of Inositol in Eukaryotes

The approach based on information granulation has identified critical boundaries and captured meaningful differences among organisms [1]. In a previous paper, we performed an analysis of the metabolic networks over a set of living organisms (5299), reconsidering the relevance of taxonomic factors [2]. The first striking divergence, the most ‘fundamental’ division of life, occurs between eukaryotes and prokaryotes and emerges in the cellular architecture of each cluster. These differences can be traced back to a few significant ‘signature’ edges, which correspond to a limited set of biochemical reactions. It is worth nothing that all of these reactions involve inositol and its principal metabolic pathways.

Inositol found in prokaryotes is mostly committed to preserving physiological osmolarity values and a correct acid–base balance [3]. Inositol in eukaryotes displays a bewildering number of functions, not only as myo-inositol (myo-Ins) but also as a building block of several related metabolites (inositol phosphates, phosphoinositides, and pyrophosphates) that are involved in multiple signaling pathways. Namely, the presence of phosphatidyl-inositol phosphates in the membranes of cells [4], nuclei [5], and membrane-bound organelles (e.g., endoplasmic reticulum, Golgi complex, endosomes, and lysosomes) strongly suggests that inositol-conjugated molecules play a critical role in the crosstalk between cells and their environment. 

The nuclear and cell membranes are the ‘gates’ intermediating pivotal signaling pathways. Their molecular substrate interfaces cells and the environment, and exerts its constraints and driving effects upon biological functions [6]. The eukaryotic organization makes membranes the favored site of signal exchange between cells and the external milieu. Furthermore, many differentiating pathways arise from the specialized structures formed by cell-to-cell and cell–substrate interactions [7]. 

Within or nearby those structures, some complex molecules play a critical role as “transducers” of a number of stimuli. It is remarkable that all of these structures involve ‘inositol-linked’ signature edges. Furthermore, the reshaping of cell architecture from prokaryotes to eukaryotes appears to have pivoted upon a very versatile, ‘simple’ molecule such as inositol. Therefore, inositol looks similar to the evolutionary ‘tipping point’ that enabled a global critical transition, as was to be expected for complex organisms governed by non-equilibrium thermodynamics [8,9]. Noticeably, the inclusion of inositol into membranes allows for an intermingled ‘compartmentalization’ of different processes [10], thus adding greater complexity to the overall system. 

Inositol also plays a basic role in the regulation of several biochemical pathways in pathological conditions such as psychiatric syndromes, diabetes, metabolic disorders, cancer, morphogenesis defects, and fertility-related diseases [11]. The discovery of its influence on endocrine signal transduction has contributed to ameliorating treatments of numerous endocrinological and gynecological syndromes [12].

Herein, we have provided a review of the last three decades of publications devoted to this fascinating area of study.

### Search Strategy

We used Google, Scopus, Web of Science, PubMed, and CAB abstract to identify studies published between 1990 and 2022 based on six keywords (see Table 1). Then, we screened these papers for scope relevance, inclusiveness of information, and quality of the research. A further search was performed to identify hard copies of the library and scientific community exchanges. Here, we selected all the papers that addressed the following question: “Does inositol and its isomers influence or modify endocrine signaling and steroidogenic pathways in the mammalian ovary?”.

## 2. Basics of Inositol

Inositol history dates back to 1850, when Johann Joseph Scherer extracted a hexa-hydroxy-cyclohexane compound from muscle cells. This is the reason for the name “inositol”—from the Greek term “is” (genitive “inos”), which means “muscle” [13]. Only later was the cyclohexanol structure established, although the precise conformation of isomers has only been described in recent times [14]. The basic hexahydroxycyclohexane backbone allows for the establishment of nine isomers (*cis*-, *epi*-, *allo*-, *myo*-, *neo*-, *scyllo*-, *L-chiro*-, *D-chiro*- and *muco*-inositol). Myo-Ins is the most abundant form in nature [15]. Scientific evidence accumulated over the past 30 years has shown that a few of those isomers (including myo-, D-chiro- and scyllo-inositol) play a significant biological and medical role. Nonetheless, despite the great achievements over the previous decades, the specific role sustained by inositol hexakisphosphate stereoisomers in living organisms (as well as in terrestrial and aquatic ecosystems) is yet to be understood [16]. Myo-Ins can be converted into D-chiro-inositol under the enzymatic activity of tissue-specific epimerases. Epimerases are activated in response to insulin; however, some other factors can participate in modulating tissue-sensitivity, probably according to specific needs. Indeed, D-chiro-Ins concentrations show relevant dissimilarities between different tissues, and the conversion rate of myo-Ins to D-chiro-Ins ranges from 3% to approximately 9%, as measured by the analysis of radiolabeled [3H]-myo-Ins. In contrast, the production of other isomers is minimal and did not exceeds 0.06% of the total radiolabeled myo-Ins^87^.

In humans, approximately 1 g/day of myo-Ins is supplied by alimentary consumption, especially through the consumption of cereals, legumes, seed oils, and nuts. However, a large fraction of myo-Ins is synthesized endogenously—from 1 to 4 g/day, depending on the specific requirements and diet [17].

Myo-Ins is synthetized endogenously from glucose-6-phosphate (G6P), which is then subject to epimerization, leading to inositol-3-phosphate (Ins-(3)-P) by D-3-myo-inositol phosphate synthase (inositol synthase, MIPS1) [18]. This is encoded in mammals by the inositol-3-phosphate synthase 1 (ISYNA1) gene [19]. Then, through inositol monophosphatase-1 (IMPA1 or IMPase), Ins-(3)-P is dephosphorylated into free myo-Ins [20]. The ISYNA1 gene is essential for normal mammalian replication and differentiation, as knockout cells cultured in inositol-free media were unable to proliferate. In animals, ISYNA1^−/−^ showed a dramatic modification in the global gene expression profile and a significant increase in embryonic lethality [21]. In yeasts, the environmental availability of inositol controls the transcription of the INO1 gene (the homolog of MIPS1) through the Ino2/Ino4 complex, which senses cellular inositol depletion [22]. Noticeably, in mammalian cells, ISYNA1 transcription is largely independent of extracellular availability of inositol as the gene relies mainly on internal cellular signals [23]. Indeed, activation of AMPK downstream of reduced intracellular glucose concentrations—altogether with increased intracellular release of phosphatidic acid (PA) from cell membranes—significantly impairs ISYNA1 expression, leading to reduced levels of MIPS1 [24]. It is worth noting that this effect is mostly dependent on the nuclear translocation of IP6K1—the enzyme that catalyze the transformation of inositol hexakisphosphate (InsP6) into diphosphoinositol pentakisphosphate (InsP7/PP-InsP5) [25]. Intriguingly, IP6K1 senses changes in the ATP/ADP ratio, and it is involved in cancer and metabolic regulation.

Those findings suggest that endogenous inositol homeostasis is closely linked to glucose metabolism and its synthesis is regulated accordingly; inositol production can be reduced if the glucose requirement increases, as happens in several physiological and pathological conditions, including cancer.

## 3. Inositol as an Intermediate Transducer of Endocrine Signals

Myo-Ins constitutes the building block of several derived inositol phosphates (InsPs), complex macromolecules such as phosphatidyl inositol (PI), its seven derived phosphoinositides (phosphatidyl inositol phosphate, PIP), and the glycosylphosphatidylinositol-anchored proteins (GPI) that are mostly localized on cell membranes [26]. This complex and interrelated network system is likely organized to cope with the challenges of an always-changing milieu. In order to efficiently cope with and react to these changes while preserving the homeostasis of living architecture, cells have established an elaborate network of molecular and biophysical “countermeasures”. It is worth noting that the plasticity of inositol-derived molecules, simply managed by the covalent attachment of phosphate groups (PO3^−^) to the inositol ring, affords a huge array of structures, which can be used sparingly by these signaling processes [27]. The importance of the inositol complex signaling network has been already investigated in plant physiology. We are only at the beginning in appreciating its relevance in mammals [28].

**Figure 1:**
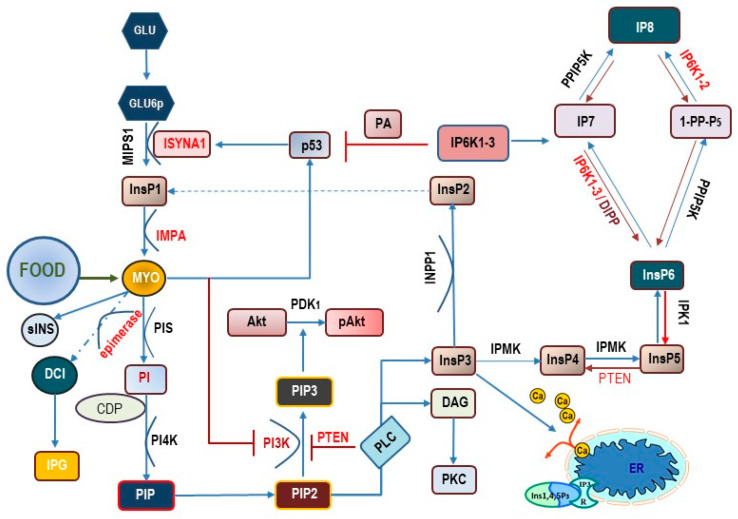
Main metabolic pathways related to myo-inositol transformation inside the cell. Myo-Ins is obtained from external sources (food) and produced by a biosynthetic pathway departing from glucose (Glu) and glucose-6-phosphate (Glu6p). Under the catalytic activity of myo-inositol-1-phosphate synthase 1 (MIPS1)—encoded by the ISYNA1 gene—Glu6p is converted into inositol-1-phosphate (InsP1) and then dephosphorylated to myo-Ins under the action of inositol monophosphatase-1 (IMPA). Myo-Ins activates p53 synthesis, which in turn reinforce ISYNA1 expression, according to a positive regulatory loop. Myo-Ins can be converted into scyllo-Ins (sINS) or D-chiro-inositol (DCI) under the action of an insulin-dependent, tissue-specific epimerase. D-chiro-Ins and myo-Ins are incorporated as inositol phosphoglycan (IPG) in glycosylphosphatidylinositol-anchored proteins (GPI). Myo-Ins can be converted into phosphatidylinositol (PI) under the activity of phosphatidylinositol synthase (PIS) in presence of CDP-diacylglycerol—inositol 3-phosphatidyltransferase (CDP). The intervention of phosphatidyl-inositol kinase-4 (PI4K) leads to the synthesis of phospathidyl-inositol-4,5-phosphate (PIP2). PIP2 is transformed by phospholipase-C (PLC) into 1,2-diacylglycerol (DAG)—that in turn activates protein kinase C (PKC)–and 1,4,5-trisphosphate (InsP3). By binding to specific InsP3-receptors, InsP3 induces calcium release from the endoplasmic reticulum (ER). PIP2 can be converted to phosphatidylinositol- 3,4,5 –trisphosphate (PIP3) by the activity of the class I phosphoinositide 3-kinases (PI3K). PIP3 formation is antagonized by the phosphatase and tensin homolog (PTEN), which acts by removing a phosphate group. PIP3 promotes the recruitment of (Akt) close to the membrane where the activity of 3-phosphoinositide-dependent protein kinase-1 (PDK1) catalyzes the phosphorylation of protein kinase B (PKB, also known as Akt) to produce pAkt. InsP3 can be de-phosphorylated by inositol polyphosphate 1-phosphatase (INPP), yielding inositol-2-phosphate (InsP2) and probably inositol-1-phosphate (InsP1). Alternatively, InsP3 can be phosphorylated by inositol polyphosphate multikinase (IPMK) into inositol-4-phosphate (InsP4), inositol-5-phosphate (Insp5), and finally, into inositol-6-phosphate (InsP6). From here, the sequential intervention of other kinases (inositol hexakisphosphate kinase 1-2-3, IP6K1-2-3; in yeast, DIPP = diphosphoinositol polyphosphate phosphohydrolase; inositol hexakisphosphate and diphosphoinositol-pentakisphosphate kinase, PPIP5K) several pyrophosphates (diphosphoinositol pentakisphosphate, IP7 and bis-diphosphoinositol tetrakisphosphate, IP8) are produced. Noticeably, IP6K1 inhibits p53.

Myo-Ins is incorporated into eukaryotic cell membranes as phosphatidyl-myo-inositol. Hence, the inositol ring can be phosphorylated by different kinases on the three, four, and five hydroxyl groups in seven different combinations, leading to three main phosphoinositides: PIP1, PIP2, and PIP3. Phospathidyl-inositol-4,5-phosphate (PIP2) plays a very critical function because phospholipase C (PLC) can split PIP2 into 1,2-diacylglycerol (DAG) and 1,4,5-trisphosphate (InsP3). The latter is a key second messenger which, by binding to specific InsP3-receptors, induces calcium release from the endoplasmic reticulum [29,30]. In both plants and protozoa, myo-Ins may be directly phosphorylated by an inositol kinase to yield InsP3 [31]. However, in humans, InsPs are initially obtained from the hydrolysis of phosphoinositides, and downstream of the dephosphorylation of higher phosphorylated forms (including InsP6) by specific phosphatases. A critical transition point in inositol intracellular metabolism is therefore represented by the hydrolysis of PIP2 to release DAG and InsP3. Of note, InsP3 has a short half-life within the cell as it is rapidly metabolized through one of the two following pathways [32]: removal of the 5-phosphate from the inositol ring by Ins-polyphosphate 5-phosphatases, resulting in the release of InsP2, and successively of InsP1, before being dephosphorylated to reconstitute free myo-Ins; a very different biochemical pathway leads to the successive phosphorylation of InsP3 to produce InsP4, under the action of Ins(1,4,5)P3 3-kinase [33]. Other inositol phosphates (InsP5 and InsP6) are ultimately produced under the action of inositol 1,3,4,5,6-pentakisphosphate 2-kinase [34], even though other kinases (IPK1 and IPK2) can efficiently recapitulate InsP6 synthesis from Ins(1,4,5)P3 [35]. Recently, it has been shown that PLC-generated InsPs are rapidly recycled to inositol, while the enzyme inositol tetrakisphosphate 1-kinase 1 (ITPK1) phosphorylates Ins(3)P originating from glucose-6-phosphate, and Ins(1)P generated from sphingolipids, to synthetize InsP6 (Figure 1) [36].

To date, physiological roles have been recognized for 63 inositol phosphate esters [37], even though the fully phosphorylated InsP6 constitutes the most abundant inositol-derived phosphate. InsP6 displays several relevant activities within cells [38], and it represents the building block to which successive phosphate groups are added to yield inositol pyrophosphates (PP-IPs). Here, one or two energetic di-phosphate bonds are crowded around the six-carbon inositol ring [39].

Inositol pyrophosphates gained momentum in the last decade as scientific investigations have unveiled many basic biological functions of PP-IPs and related kinases [40] in mammals, including morphogenesis, apoptotic, and proliferation processes [41,42]. Noticeably, increasing evidence suggests that inositol pyrophosphates can play a key regulatory role in glucose and phosphate metabolism by fine-tuning the balance between glycolysis and mitochondrial oxidative phosphorylation in ATP production [43,44]. As a proof of concept, the pharmacological use of inositol has been proven to modulate some pathological conditions in a significant fashion [45].

An impressive body of evidence suggests that treatment based on myo-Ins can dramatically modify several key processes, including cytoskeleton dynamics, epithelial-mesenchymal transition, ovary steroidogenesis, oocyte maturation, and insulin resistance, just to mention a few [46,47,48]. However, it is hazardous to ascribe these effects to myo-Ins, given that its activity can depend either on secondary metabolites or on changes induced by myo-Ins addition, upon the balance among different PIs isoforms. Unfortunately, the intracellular dynamics that govern myo-Ins transformation under different physiological and pathological circumstances in mammalian cells still needs to be investigated. This is a critical issue given that significant changes in myo-Ins metabolism has been observed in different pathological conditions, e.g., PCOS, cancer, and insulin resistance [49,50], where those modifications are deemed to play a pathogenetic role.

Organisms have the ability to respond to their environment in order to adapt and survive. In response to physical stresses and biochemical signals, cells usually transduce those stimuli by means of cytoskeleton changes (mechanotransduction) and receptor activation by finally modulating a selected number of second messengers. Second messengers should rise rapidly and must disappear in a short period of time to allow a ‘discrete’ and tuned transmission of the signal. The most common module of cellular signaling pathways relies on phosphorylation/dephosphorylation processes, which are associated with activation/deactivation mechanisms. The balance between phosphorylation and dephosphorylation is indeed critical in the enzymatic activation, and it is mostly regulated by the dynamic fluctuations of specific enzymes (kinases and phosphatases), as well as of their substrates and cofactors. A simple way to turn off/on a signal can be represented by adding/removing a phosphate group, as so happens within the intense trafficking in the pool of InsPs and PP-IPs of both the cytosol and the nucleus [51]. Likewise, phosphorylation/dephosphorylation of phosphoinositides—namely the splitting of PIP2 into DAG and InsP3—represents a further mechanism through which inositol-related molecules can modulate signal transduction across the dynamical interface between cells and the microenvironment (cell membrane), as well as between the cytosol and the nucleus (nuclear membrane) [5,52]. A crucial role is sustained by PIP2, which regulates a number of crosstalks across cell/nuclear membranes, while occupying a pivotal bifurcation point that directly or indirectly controls several biochemical pathways downstream [53], including InsP3 release. Of note, InsP3 is not only instrumental in Ca^2+^ release but it is the basic brick from which myo-Ins stores can be reconstituted through successive dephosphorylating steps. On the other side, PIP2 phosphorylation mediated by the PI3K family enzymes leads to PIP3 and PIP4 formation and to the activation of a number of cascade events, including Akt phosphorylation. Furthermore, PIP2 serves as a docking support that binds specific domains promoting the recruitment of proteins to the cell membrane with subsequent activation of a bewildering number of signaling cascades [54].

## 4. D-Chiro-Inositol and Myo-Inositol

Myo-inositol participates in the modulation of wide arrays of signaling pathways through its derivatives, including inositol phosphates, pyrophosphates, phospholipids, and glycosylphosphatidylinositol-anchored proteins. However, one of its most relevant isomers, i.e., D-chiro-inositol (D-chiro-Ins), does not enter into this intricate network. Rather, it likely acts either as such or through its phospho-glycan derivatives (IPGs). Under insulin stimulation, tissue-specific epimerase enzymes convert myo-Ins into its stereoisomer D-chiro-Ins [55]. This irreversible reaction undergoes fine-tuning that leads to very different concentrations of the two isomers within different tissues [56]. Moreover, both of these inositols participate in the constitution of GPI anchors in which they represent the IPG-core. IPGs incorporating either myo-Ins (IPG-A) or D-chiro-Ins (IPG-P) are released upon the stimulation of insulin by hydrolysis of GPI lipids located on the outer leaflet of the cell membrane [57]. IPGs influence intracellular metabolic processes, especially by modulating several steps of the oxidative and non-oxidative metabolism of glucose, as well as by exerting several insulin-like actions [58]. It is worth mentioning that myo-Ins also promotes translocation to the plasma membrane of the glucose transporter GLUT4, to enhance glucose uptake, while reducing free fatty acid release from adipose tissue [59].

D-chiro-Ins enhances glycogen synthase activity, and D-chiro-Ins availability is remarkably increased in tissues with large glycogen deposits, such as the liver or skeletal muscle [60]. Moreover, while IPG-P promotes the activation of pyruvate dehydrogenase (PDH) and PDH phosphatases (PDHP), IPG-A inhibits both protein kinase A and adenylyl cyclase (AC) [61]. Moreover, IPGs increase mRNA and protein expression of the insulin receptor substrate (IRS1), PI3K, and AKT, upregulating the level of the p-AKT protein and down-regulating GSK3β protein [62]. Overall, these effects can be considered ‘insulin-mimetic’ activities [63]. Furthermore, D-chiro-Ins increases PDH activity and thus enhances the oxidative transformation of glucose along the Krebs cycle. This results in the production of adenosine triphosphate (ATP) [64]. Noticeably, ATP shortage and insulin overstimulation leads to overactivation of IP6K1, which in turn promotes the synthesis of 5-diphosphoinositol pentakisphosphate (5-IP7). This pyrophosphate reduces insulin sensitivity by preventing the interaction between Akt and PI3K and impairing GSK3β and mTOR signaling pathways, which are both associated with insulin resistance and weight gain [65]. Conversely, it has been demonstrated that the genomic deletion or enzymatic inhibition of IP6K1 reduces cell invasiveness and migration capacity, protecting against chemical-induced carcinogenesis [66]. Noticeably, myo-Ins can dramatically down-regulate IP6K1 expression by increasing ATP availability (by enhancing oxidative glucose metabolism) and through up-regulation of the miRNA 125a-5p. Consequently, myo-Ins almost completely inhibited the metastatic potential in a model of invasive breast cancer cells, while promoting a reversion of the cancerous phenotype [67].

Through all these mechanisms, myo-Ins and D-chiro-Ins may exert their insulin-sensitizing effect, thereby decreasing insulin requirements. This has been confirmed by clinical trials in which patients with insulin resistance have been treated with myo-Ins, D-chiro-Ins, or a combination of the two [68,69].

## 5. Myo-Inositol and D-Chiro-Inositol in the Ovary

**Figure 2:**
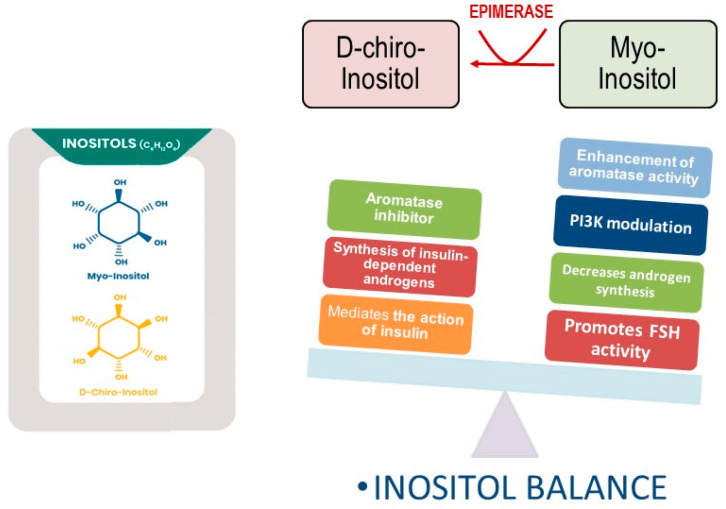
Opposite functions exerted by myo-inositol and D-chiro-inositol. D-chiro-Ins inhibits aromatase synthesis and increase androgen synthesis under insulin stimulation. Myo-Ins modulates PI3K activity, while enhancing aromatase and FSH-receptor synthesis in granulosa cells. Moreover, myo-Ins decreases androgen release from theca cells.

However, this is where the activities of the two isomers diverge. In fact, these molecules display complementary and even opposite effects upon ovary steroidogenesis (Figure 2).

First, Nestler et al. [70] observed that, in theca cells, upon insulin addition, the release of D-chiro-Ins containing phosphoglycans stimulated the biosynthesis of testosterone. Noticeably, an antibody directed against the inositol phosphoglycan containing D-chiro-Ins nullified this effect. Furthermore, it was observed that D-chiro-Ins directly regulates steroidogenic enzyme genes in human granulosa cells, reducing the mRNA expression of both aromatase and cytochrome P450 side-chain cleavage genes in a dose–response fashion [71]. Indeed, D-chiro-Ins seems to enhance the synthesis of androgens in the ovary while down-regulating estrogen release through inhibition of the synthesis of aromatase. Those findings shed light on some controversial clinical results obtained in patients with polycystic ovarian syndrome (PCOS) treated with high doses of D-chiro-Ins. Although D-chiro-Ins was able to normalize several systemic metabolic parameters related to insulin resistance, that investigation [72] could not confirm the beneficial effects upon ovary function previously obtained by using low doses of D-chiro-Ins in patients with PCOS [73]. Moreover, high D-chiro-Ins levels undesirably influence the quality of oocytes and blastocysts [74]. Conversely, treatment of male volunteers with D-chiro-Ins (1 g/day) led to a dramatic reduction in both estrone and 17β-estradiol levels (−85.0% and −14.4%, respectively), while increasing testosterone and dehydroepiandrosterone (+23.4% and +13.8%, respectively) [75]. These results suggest that D-chiro-Ins could exert appreciable anti-estrogenic activity in the treatment of male and female clinical states that would benefit from androgen increase and/or estrogen decrease [76].

On the contrary, myo-Ins supplementation (at pharmacological doses of up to 2 g/daily) demonstrated beneficial effects on PCOS subjects. Treatment with myo-Ins significantly decreases the LH/FSH ratio in the plasma of women with PCOS [77,78]. Moreover, supplementation with myo-Ins during in vitro fertilization treatments allowed for a significant reduction in the amount of recombinant FSH administered [79]. Preliminary results from in vitro studies indicate that myo-Ins increases FSH receptor and aromatase synthesis in granulosa cells, probably through a FSH-independent mechanism of action. In fact, myo-Ins also increased the transcription of aromatase in breast cancer cells, even in the absence of any previous FSH administration [80]. These results seem to indicate an opposite role of myo-Ins and D-chiro-Ins in ovarian steroidogenesis: while D-chiro-Ins down-regulates aromatase activity and increases androgen production, myo-Ins mitigates androgen synthesis and increases the expression of both the FSH receptor and aromatase. It is worth noting that the downregulation of the FSH receptor and aromatase in granulosa cells constitutes a hallmark of PCOS [81]. Therefore, it should be expected that D-chiro-Ins supplementation in this condition would worsen the clinical picture. Indeed, as demonstrated by Bevilacqua et al. [47], only treatments with high myo-Ins concentrations (and low D-chiro-Ins content) have been effective in treating PCOS in mice. In a previous study, animals were treated with different myo-Ins/D-chiro-Ins formulas (from 5:1 to 80:1), and only mice receiving myo-Ins/D-chiro-Ins in a 40:1 molar ratio experienced a prompt and complete recovery from PCOS signs and symptoms. When the treatment comprised higher D-chiro-Ins concentrations,—with myo-Ins/D-chiro-Ins ratios ranging from 5:1 to 20:1—paradoxically, the results indicated worse clinical and biochemical pathological characteristics of the syndrome. Several clinical trials have confirmed these experimental results, indicating that treatment based upon a myo-Ins/D-chiro-Ins ratio close to values normally found in the blood (40:1) are effective in managing PCOS symptoms [82]. The unexpected, paradoxical sensitivity of ovary steroidogenesis to D-chiro-Ins may be explained according to the suggestion proposed first by Unfer [83]. In physiological conditions, the myo-Ins/D-chiro-Ins ratio ranges from 40:1 in plasma to 100:1 in follicular fluid [84]. It is worth noting that, following insulin stimulation, the highest conversion rate of myo-Ins into D-chiro-Ins approached 9% in organs/tissues that are highly dependent on insulin, while in less-insulin-sensitive areas—such as the brain and heart—conversion barely reached 2% [85]. These differences highlight that the requirement of D-chiro-Ins pointedly changes according to tissue-dependent sensitivity and metabolic demands. However, insulin resistance can dramatically reduce D-chiro-Ins synthesis in insulin-dependent organs, with resulting low intracellular levels of this inositol isomer [86]. Conversely, the administration of D-chiro-Ins has proven to ameliorate several symptoms and metabolic parameters associated with diabetes or insulin resistance, henceforth decreasing insulin requirements and, consequently, the insulin-dependent stimulus on androgen synthesis [87]. However, insulin resistance is not associated with impairment of the insulin transduction signal at the ovarian level, given that hyperinsulinemia still stimulates ovarian androgen production in PCOS [88]. Therefore, insulin increases the ovarian myo-Ins conversion into D-chiro-Ins. The resulting increase in D-chiro-Ins levels induced by insulin (or obtained following the administration of the isomer at high concentrations) impairs ovarian availability of myo-Ins, as demonstrated by Larner’s seminal paper [89], determining an imbalance in the myo-Ins/D-chiro-Ins ratio. Consequently, D-chiro-Ins, while ameliorating the PCOS-related systemic metabolic parameters, exacerbates abnormal steroidogenesis within the ovary, thus providing a mechanistic rationale that supports the so-called Unfer paradox [47]. Those findings also help to explain why treatment with antidiabetic drugs (metformin) improves several PCOS-associated metabolic markers [90] (including androgen production) but is still scarcely effective in ameliorating ovarian function. This is witnessed through the recorded decrease in follicle number and quality [91]. Conclusively, insulin resistance alone as a metabolic disorder cannot explain the complexity of PCOS. It is wrong to assume a PCOS pathogenesis model in which the metabolic “defect” (i.e., defective insulin transduction due to the impaired availability of inositol phosphoglycans) plays a causative role. Indeed, this framework underestimates the steroidogenic effects directly triggered by D-chiro-Ins. Consequently, normalizing the myo-Ins/D-chiro-Ins ratio would be beneficial in restoring physiological ovarian function, even in the absence of insulin resistance. This point has been vindicated by clinical trials performed in lean and young women with PCOS but without insulin resistance, where myo-Ins based treatment still retains its efficacy [92,93]. Therefore, we would suggest that the primary pathogenetic defects in PCOS could be an intrinsic altered androgen synthesis within the ovary, eventually complicated by other systemic factors (e.g., insulin resistance, deregulation of the hypothalamic–hypophysis axis, and obesity), with subsequent reduced FSH sensitivity, aromatase levels, and estradiol bioavailability [94]. Within this model, the altered balance between myo-Ins and D-chiro-Ins, resulting in a relative excess of the latter component, may represent a causative relevant factor. Indeed, analyses of the follicular fluid obtained from women with PCOS have recorded a significant decrease in the myo-Ins/D-chiro-Ins ratio; a while normal myo-Ins/D-chiro-Ins proportion is nearly 100:1, in the follicular fluid of women with PCOS, that value decreases to 0.2:1 [84]. Reduced intra-ovarian availability of myo-Ins may also explain why a fraction of PCOS cases (30–40%) are “refractory” to myo-Ins based treatments, given that this “resistance” can be ascribed to insufficient inositol uptake. Indeed, when co-administration of α-lactalbumin (that reversibly opens the tight junctions) [95] significantly improves inositol absorption from the gut, ovulation is restored in women with PCOS, and several PCOS features recover following myo-Ins treatment [96].

**Figure 3:**
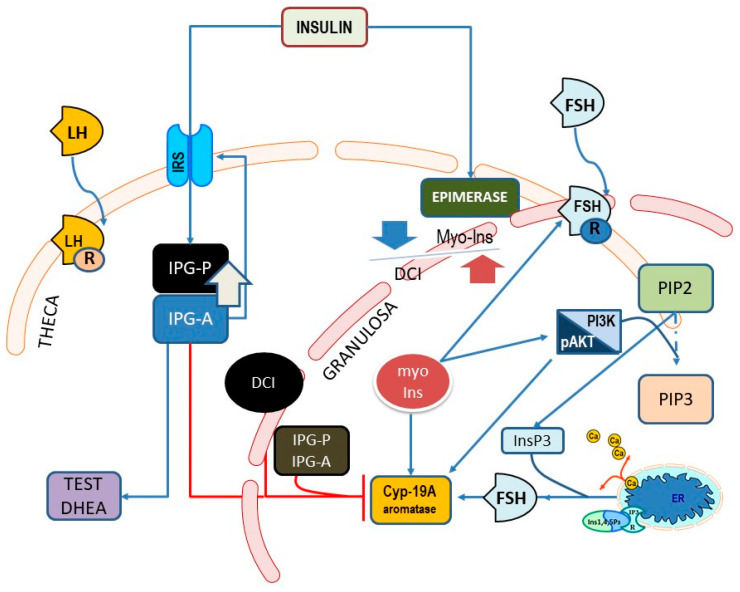
Main pathways showing the involvement of myo-Ins and D-chiro-Ins (DCI) upon endocrine signaling pathways in theca and granulosa cells within the ovary. Cyp-19A, aromatase; β-CAT, β-catenin; InsP3, inositol-e-phosphate; ER, endoplasmic reticulum; IP3R, InsP3-receptor; FSHr, FSH receptor; IPG, inositol phosphoglycan containing either D-chiro-Ins (P) or myo-Ins (A); IRS, insulin receptor substrate (IRS) proteins.

To sum up, both myo-Ins and D-chiro-Ins remarkably modulate insulin transduction and glucose metabolism, while exerting an opposite role upon ovarian steroidogenesis (Figure 3). This highlights how relevant a nutritional component can be in fine-tuning a critical endocrine network.

## 6. Conclusions

Inositols participate in the endocrine control of ovarian function by modulating the signaling of a number of critical hormones, including FSH and insulin. Namely, it has been observed that D-chiro-Ins favors the transduction of insulin signal and modulates androgen release, while inhibiting aromatase synthesis in the ovary. On the contrary, myo-Ins mainly promotes both FSH responsiveness and aromatase synthesis. While D-chiro-Ins likely exerts its effect by itself or, ultimately, through inositol phosphoglycan (IGP-P), myo-Ins is the starting block of several relevant metabolites that display a plethora of biological functions. Hence, assigning a causative role to myo-Ins in the cell’s complex signaling network is a hard task. To obtain a reliable picture, we should be able to capture the overall metabolic fate of myo-Ins when added to a specific tissue/cellular context, and to correlate changes in inositol metabolism with the associated biochemical pathways. Fertility and cancer research, for example, showed some progress in that sense but it is still a long way from grasping the basic interaction mechanisms of inositol. However, emerging evidence indicates that myo-inositol, and its isomers and related metabolites provide for a complex network of ‘signaling’ molecules. These are particularly involved in endocrine signal transduction, which frames inositol alterations within endocrinological diseases.

Unfortunately, the analytical assessment of myo-inositol phosphates and myo-inositol pyrophosphates is a challenging task as we are faced with a high number of isomers, often at very low detectable levels, with different charges. Furthermore, the chemical instability of the derived compounds (namely esters, and hydroxylated and anhydride derivatives) complicates the analytical assay even further [97]. However, the analytical “recognition” of the whole map of inositol-related metabolites is needed in order to reconstruct the overall signaling network. The complexity of inositol metabolism has, in fact, severely constrained the possibility of making substantial advancements in the field. Among the most reliable technology currently available, the one preferentially used is based on metabolic labeling with tritiated [3H]-inositol, followed by acidic extraction, strong-anion-exchange high-performance liquid chromatography (SAX-HPLC), and manual scintillation counting of the selected parts. However, this is a time-consuming and expensive strategy, requiring specialized radioactive instrumentation and laboratories. In addition, it cannot provide any useful information regarding myo-Ins endogenously generated from D-glucose-6-phosphate by ISYNA1. As a consequence, techniques based on post-column-derivatization UV detection have been proposed as an alternative to radiolabeling [98,99]. Furthermore, several other strategies are currently under investigation to allow a reliable assessment of myo-Ins and its metabolites [100]. Improved analytical techniques are required to address a number of critical questions, such as those recently raised by Saiardi [17]. First, why does ISYNA1 display specific patterns of expression in each tissue, and why are the early steps of morphogenesis (not restricted to the embryo) so sensitive to the consequences associated with ISYNA1 abrogation? These pivotal questions can help to explain the role played by myo-Ins in several morphogenetic processes, not just those limited to physiological processes, but also encompassing the development of cancerous tissues. Second, according to the compartmentalization model [8], does the cell metabolize myo-Ins that is endogenously produced and that absorbed from nutrients differently? This questions whether these two “different” inositols can be directed toward selective biosynthetic pathways: one leading to phospho-inositol phosphates and the other to inositol phosphates and pyrophosphates. An approach in which new analytical techniques, such as HILIC-MS/MS and CE-MS, are coupled with molecular biology can provide priceless insights into the complex signaling network supported by inositol and its metabolites.

## Figures and Tables

**Table 1:** Search review criteria.

Number	Keywords	Years
1	Boolean logic such as “AND”, “OR”, “NOT” were used	1990–2022
2	Myo-inositol	
3	D-chiro-inositol	
4	Inositol phosphates	
5	Inositol pyrophosphates	
6	Phosphoinositides	
7	Endocrine signaling	

## Data Availability

The data presented in this study are available in the article.
